# Secondary prevention of acute coronary syndrome with antiplatelet agents in real life: A high-dimensional propensity score matched cohort study in the French National claims database

**DOI:** 10.1016/j.mex.2020.100796

**Published:** 2020-01-23

**Authors:** Patrick Blin, Caroline Dureau-Pournin, Jérémy Jové, Regis Lassalle, Cécile Droz, Nicholas Moore

**Affiliations:** Affiliations: Bordeaux PharmacoEpi, INSERM CIC 1401, University of Bordeaux, France

**Keywords:** Real-life performance of drugs, High-dimensional propensity scores, Cardiovascular prevention, High-dimensional propensity score matched cohort study

## Abstract

Users of newly marketed drugs often differ from the patients included in randomized clinical trials, and from patients prescribed similar drugs. Cohorts of such users may be compared using propensity score adjustment, or similar user cohorts may be built using high-dimensional propensity score matching in large population databases. One such database is SNDS, the French nationwide claims and hospitalization database, which covers 99 % of the French population. It has yet been rarely used.

To study the comparative effectiveness and safety in secondary coronary prevention of ticagrelor, compared to clopidogrel or prasugrel, we identified in SNDS patients who were dispensed any of the three antiplatelet agents of interest (± aspirin) within a month after discharge from hospital for acute coronary syndrome (ACS) and followed them one year for recurrence of ACS, stroke, acute bleeding, or death. High-dimensional propensity scores were developed to identify matched cohorts. Drug performances were also compared in the whole population using adjustment on the same parameters. Here we describe the database that was used, and the methods developed for the high-dimensional propensity score matching, resulting in standardized mean differences between the matched populations of less than 2 % for all of the 500+ variables included in the model.

• *This study was done in a newly available large-scale claims database, which may differ from other population databases, by it size and exhaustiveness*
• *The methods elaborate on standard high-dimensional propensity scores as adapted to this claims database*

## Graphical abstract

**Figure:**
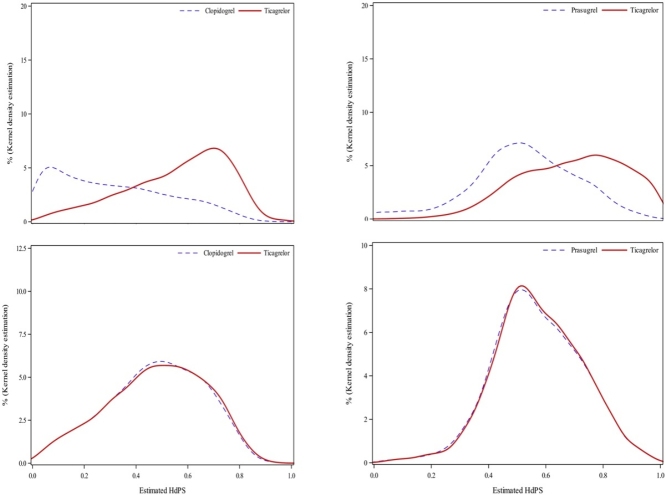

Specifications Table

**Table:** 

Subject area:	Pharmacology, Toxicology and Pharmaceutical Science Clinical sciences
More specific subject area:	Pharmacoepidemiology Drug safety Comparative effectiveness research Epidemiology
Method name:	High-dimensional propensity score matched cohort study
Name and reference of original method:	Rassen JA, Glynn RJ, Brookhart MA, Schneeweiss S. Covariate selection in high-dimensional propensity score analyses of treatment effects in small samples. Am J Epidemiol. 2011;173(12):1404-13 [1].
Resource availability:	NA

## Method details

We describe here the methods used to compare the effectiveness of antiplatelet agents in secondary prevention after an acute coronary syndrome [2].

The originality of this study is related to

- the use of the French National single-payer healthcare database SNDS, which has only recently been made available for researchers. This allows for large population sizes and representativeness (SNDS covers 99 % of the French population from birth to death) [3].

- the use of high-dimensional propensity score matching, made possible by the large number of subjects and variables in the database, resulting in near-identical comparative cohorts. This study builds on previously published methods, that were developed in the different context of mostly North American claims databases [1,[4], [5], [6]].

The present paper presents the general study design, the database that was used, the variables and the methods used for the development of the propensity score, as well as the results of matching on the distribution of population variables between the unmatched and matched cohorts

### Research questions

The research question was to evaluate in real-life the use and the impact of Ticagrelor and other antiplatelet agents (APA) in the secondary prevention of acute coronary syndrome (ACS).

### Objectives

#### Main objective

The main objective was to estimate the one-year incidence of the primary effectiveness endpoint (all-cause death or hospitalisation for ACS or hospitalisation for ischemic or undefined stroke) and the one-year incidence of the primary safety endpoint (hospitalisation for major bleeding) during Ticagrelor exposure and during other APA exposure for secondary prevention of ACS.

#### Secondary objectives

Secondary objectives are:

- to estimate the one-year incidence of the secondary effectiveness endpoint (all- cause death or hospitalisation for ACS or hospitalisation for percutaneous coronary intervention (PCI) or coronary artery by-pass grafting (CABG) or hospitalisation for ischemic or undefined stroke) during Ticagrelor exposure and during other APA exposure for secondary prevention of ACS;
- to estimate the one-year incidence of the secondary effectiveness endpoint (all- cause death or hospitalisation for ACS in intensive care or hospitalisation for ischemic or undefined stroke) during Ticagrelor exposure and during other APA exposure for secondary prevention of ACS;
- to describe the characteristics of patients hospitalised for ACS according to APA treatment prescribed after discharge;
- to describe conditions use of APA treatments (dosage and number of boxes dispensed, number of dispensings, other treatments for ACS secondary prevention, and concomitant treatments) over one year;
- to estimate the retention rate of APA treatment, the frequency of treatment discontinuation over one year, and the frequency of switches;
- to compare the one-year incidence of the primary and secondary effectiveness endpoints between APA during secondary prevention of ACS, taking into account patients’ and index ACS characteristics, as well as other drugs for ACS secondary prevention and physician follow-up (mainly cardiologist or not);
- to compare the one-year incidence of the primary safety endpoint between APA during secondary prevention of ACS, taking into account patients’ and index ACS characteristics, as well as other drugs for ACS secondary prevention and physician follow-up (mainly cardiologist or not).

### Research methods

#### Study design

The design is a historical cohort study in a national healthcare claims and hospitalisations database, SNDS, including patients hospitalised in 2013 for an ACS with one-year previous history and at least one year follow-up in the database.

Data are extracted from 1 st January 2012 to 31 st December 2014:

- the index date is the date of discharge of the first hospitalisation for an ACS between 1 st January 2013 and 31 st December 2013;
- the study follow-up period starts on the study index date and ends one year later.
- Each patient has a one-year history before the index date and at least one year of follow-up. 

#### Subjects

##### Extraction criteria

All patients hospitalised in 2013 for an ACS, regardless of the type of treatment (medically managed, managed with PCI or CABG) were identified in the SNDS. Data from 1 st January 2012 to 31 st December 2014 were then extracted for these patients.

##### Study populations

Among these hospitalisations, those with the following criteria were selected for the analysis:

- first hospitalisation in the year 2013; 
- start and end date of index hospitalisation for ACS between 1 st January 2013 and 31 st December 2013; 
- without history of ACS in the 30 days prior to start date of index hospitalisation; 
- index hospitalisation in a hospital, teaching/regional hospital or private hospital;
- index hospitalisation duration more than zero days;
- adult patients (aged18 years or more)
- with one-year database history before the index ASC hospitalisation;
- alive at discharge of index hospitalisation;
- with at least one year of follow-up in the database after index hospitalisation (deaths excluded);
- without stay in a rehabilitation centre in the 30 days after index ACS hospitalisation (no information on drug utilisation in rehabilitation centre.

Patients who were dispensed any of the three study drugs within "à days of discharge were followed for a year (or until death). Other patients were simply described [2].

#### Study criteria

##### Inclusion disease

The analysis concerns all patients hospitalised in 2013 for an index ACS, primary diagnosis ICD-10 codes I20.0 for unstable angina and I21 for acute myocardial infarction (MI) (International classification of disease, 10th revision (ICD-10) codes listed in Appendix A).

##### Exposure definitions

- index date : date of discharge of the first hospitalisation for an ACS, used for inclusion.;
- The first dispensing of an APA (Clopidogrel, Prasugrel, Ticagrelor) in the 30 days after index date defines the treatment group; Patients with a first dispensing of APA beyond the 30 days after discharge (with or without Acetylsalicylic Acid (ASA)) were not included in this study.
- duration of drug dispensing was estimated using the number of packs of drug dispensed, the number of units per pack and the defined daily dose (DDD) for the drug;
- discontinuation of initial APA treatment defined as no dispensing of the drug during the duration of the drug supply plus the grace period of 30 days (taking into account last dispensing and the supplies of medications of the previous dispensings); Date of discontinuation was considered as date of last dispensing plus supply in DDD.
- switch of initial APA treatment defined as a dispensing of another APA (Clopidogrel, Prasugrel or Ticagrelor);
- retention of initial APA treatment defined as the period from first dispensing to the time of switch or discontinuation of initial APA treatment;
- date of APA withdrawal defined as the retention date of initial APA treatment, defined as the earlier of date of APA discontinuation as defined above, or date of APA switch;
- APA exposure defined as the period starting at the index date and ending at the end of follow-up for patients without APA withdrawal, else at the date of APA switch or discontinuation;
- APA compliance defined as the drug Medication Possession Ratio (MPR) during drug exposure defined by the number of defined daily doses (DDDà dispensed, divided by the number of days of drug exposure for APA.

##### Outcomes

Study Outcomes are outcomes that occur during the drug exposure period:

- all-cause death (cause of death not available in the database);
- hospitalisation for ACS defined as a hospital-discharge summary with primary diagnosis ICD-10 codes I20.0 (for unstable angina) or I21 (for MI);
- hospitalisation for ACS in intensive care defined as a hospital-discharge summary with primary diagnosis ICD-10 codes I20.0 (for unstable angina) or I21 (for MI) and a stay in an intensive care unit for this hospitalisation;
- hospitalisation for an ischemic or undefined stroke defined as a hospital-discharge summary with a primary diagnosis ICD-10 code listed in Appendix B;
- hospitalisation for an PCI or CABG defined as hospitalisation with *Classification Commune des Actes Médicaux* (CCAM, French medical classification for clinical procedures) codes listed in Appendix D.
- primary effectiveness endpoint (composite criterion) defined as the occurrence of the first of the, following events, during APA exposure: hospitalisation for ACS, hospitalisation for an ischemic or undefined stroke or all-cause death;
- secondary effectiveness endpoint (composite criterion) defined as the occurrence of the first following event during APA exposure period: hospitalisation for ACS, hospitalisation for an ischemic or undefined stroke, hospitalisation for PCI or CABG or all-cause death;
- secondary effectiveness endpoint (composite criterion) defined as the occurrence of the first following event during APA exposure period: hospitalisation for ACS in an intensive care unit, hospitalisation for an ischemic or undefined stroke or all-cause death;
- primary safety endpoint defined as the first hospitalisation for a major bleeding, including hemorrhagic stroke (primary diagnosis ICD-10 codes listed in Appendix C) during APA exposure period.

##### Confounding

The choice of APA could be in relation with patient, history or ACS characteristics. These characteristics have been compared at index date between treatment groups of interest (Ticagrelor vs. Clopidogrel, Ticagrelor vs. Prasugrel), and taken into account in comparisons (using both adjustment and matching).

The potential confounders were:

- Gender, age and coverage by CMU-c at index date;
- Diagnosis at the index hospitalisation (STEMI, NSTEMI, Unstable angina);
- PCI or CAGB during index hospitalisation;
- Stay in an intensive care unit during index hospitalisation;
- Category of the hospital of the index hospitalisation;
- History of ACS defined as hospitalisation for ACS in the 365 days prior to index date;
- History of APA treatment defined as drug (ASA, Clopidogrel, Prasugrel and Ticagrelor) dispensings in the 365 days prior to index date;
- History of long-term disease (LTD) prior to index date; 
- history of hospitalisation in the 365 days prior to index date; 
- history of drug dispensings in the 365 days prior to index date; 
- cardiac risk factors and other comorbidities in the 365 days prior to index date;
- Charlson comorbidity index at index date;
- probability to be treated by APA (Clopidogrel vs. Ticagrelor, Prasugrel vs. Ticagrelor), estimated using high-dimensional propensity score (hdPS), taking into account all informations of the database, with multiple data dimensions from patients and disease characteristics (LTD, one-year history of hospitalisation (primary, linked, or associated diagnosis), one-year history of drugs dispensed and one-year history of healthcare reimbursement (medical or para-medical visits and lab tests).

Index diagnosis was a real confounder. So, analysis were conducted according to three subtypes of diagnosis:

- unstable angina (UA, I20.0 ICD-10 codes); 
- ST-segment elevation MI (STEMI, I21.0, I21.1, I21.2, I21.3, I21.9 ICD-10 codes);
- non-ST-segment elevation MI (NSTEMI, I21.4 ICD-10 codes).

#### Bias

##### Selection bias

Since all patients identified with extraction criteria in 2013 were extracted from a national database, there was no study selection bias and very little attrition (emigration).

##### Information bias

SNIIRAM has the advantages of extracting patient records from an existing database, which are not impacted by study conduct:

- hospitalisation for ACS: the index hospitalisation for ACS was defined using the primary diagnosis (ICD-10 codes I20.0 or I21) at discharge in order to exclude ACS coding for suspicion or exploration, as well as miscoding for another disease. A sensitive definition was defined including all cardiac revascularisations and a specific definition including only ACS with intensive care stay during hospitalisation;
- APA exposure: APA exposure was assessed using the first dispensing after discharge and exhaustive non-hospital drug claims. Consequently, this analysis did not take into account APA switch before discharge, but the proportion of patients concerned should be low and the impact over one year would have been mainly in relation with the APA prescribed for a long time;
- outcomes: since deaths are recorded in the database using the national death registry, there is no information bias for this outcome. Clinical events were defined using the primary diagnosis (ICD-10 code) at discharge. As for the index hospitalisation for ACS, miscoding cannot be excluded but had to be sparse for the clearly defined events studied. Nevertheless, the PMSI coding is fully independent from the study and there is no reason that a potential miscoding is different between APA drugs, excluding an information bias;

##### Confounding bias

The choice of APA can be in relation with patient, his history or the characteristics of the index hospitalisation for ACS. These characteristics were compared at inclusion between APA, and taken into account in comparisons using both adjustment and matching (1:1) on gender, age, ASA at dispensing index, exposure to at least one of the four recommended drugs after an ACS (beta-blockers, ASA, statins, angiotensin- converting enzyme inhibitors (ACEI) or angiotensin receptor blockers (ARB)) during initial APA exposure period, and probability of receiving APA (using hdPS method).

##### Competing risks

Competing risks arise when individuals are subject to a number of potential failure events and the occurrence of one event might preclude the occurrence of other events. For example, death for another cause than ACS is a competing risk for ACS.

In this study, death was not considered as a competitive risk for clinical outcomes because deaths are mostly a cardiovascular event in the first year post-ACS.

##### Immortal time bias

Immortal time bias arises when time before actual exposure is included in the exposed time. In this case, there was no immortal time bias: follow-up started at the time of the first dispensing of an APA. Patients who died without any dispensing of APA were not included in the study.

## Data source

The study was done in SNDS, the French National healthcare system database. This database includes 99 % of the French population, from birth or immigration to death or emigration [3].

The SNDS (SNIIRAM) database is the national healthcare insurance system database linked to the national hospital-discharge summary database (*Programme de Médicalisation des Systèmes d'Information*, PMSI) and the national death registry. It includes the main healthcare insurance systems (CNAMTS including civil servants and students, MSA, and RSI plus secondary minr systems,), which represent 99 % of the French population. Non-hospital healthcare data are available since 2003 for CNAMTS and 2011 for MSA and RSI. Hospital discharge information is available since 2005 for CNAMTS and 2010 for MSA and RSI populations. Non-hospital data are updated every month and hospital- discharge summaries yearly at end of Q3 for the previous year.

The SNIIRAM contains individual anonymous information on: 

- General characteristics: gender, year of birth, *Couverture Mutuelle Universelle-complémentaire* (CMU-c) status, residence area;
- Date of death for those concerned; Causes of death are not available at this time but will be in the near future.
- Long-term disease with full insurance coverage (LTD and their associated ICD-10 codes) with start and end date of LTD. There are 30 LTD, which include most chronic diseases with long term and/or expensive treatment, e.g. LTD 13 is for coronary heart disease. Registration with an LTD is obtained at the request of a patient’s general practitioner and must be validated by the health insurance system physician. Once registered, patients receive full (100 %) reimbursement for expenditures related to the LTD, as defined by the health authorities. The LTD is specific for the diagnosis with a very low risk of false positives, but not sensitive because not all patients with the disease ask to benefit from a LTD. Each LTD is subdivided into the relevant ICD-10 codes (3200 ICD-10 codes included). These will be used for the identification of background or pre-existing diseases or risk factors. Because there may be a lag between the onset of the disease and its acceptance as an LTD, they are not practical for the identification of study outcomes, which rely on tracer medicines, procedures, hospital diagnoses or combinations thereof.
- Outpatient reimbursed healthcare expenditures with dates (prescription and dispensing) and codes (but not the corresponding medical indication nor result): visits, medical procedures, lab tests, drugs and medical devices;
- Pharmaceuticals are dispensed as individual pharmaceutical preparations of fixed-quantity boxes. The information includes the International non-proprietary name (INN)(e.g., ticagrelor) and the commercial name of the drug (e.g., Brilique®), as well as its unique registration number that identifies each preparation including the drug dosage and quantity of drug (number of tablets) dispensed per box, in addition to the ATC and EPhMRA codes [7,8]. The exact quantity of medication that each patient receives can be calculated, as well as the dispensing pattern. From this information daily doses can be calculated if the dispensing is repeated, especially for chronic use drugs such as those used for secondary cardiovascular prevention. The indication for the drug is not mentioned, but can usually be derived from the drug's authorized indication, patient characteristics such as age, sex and LTD, concomitant medication, specialist visits and lab tests done [9].
- Lab tests and other procedures are recorded, but not their results. In some cases the results may be presumed from actions taken after the lab tests or procedures e.g., a test for H. pylori followed by HP eradication therapy will probably indicate that the test was positive; Liver function tests or enzymes followed by hospital admission for acute hepatotoxicity will probably have been abnormal; A home intervention by a nurse shortly after the dispensing of an injectable drug will probably indicate its injection by the nurse. Of course all these are conjectural but rely on medical knowledge and common sense.

These data also include cost, so that medical costs from the insurer's viewpoint can be derived relatively easily. Overall yearly cost per patient may also be used as an indicator of general health.

- Hospital-discharge summaries from Hospital discharge summaries: ICD10 diagnosis codes (primary, linked and associated diagnosis) for all medical, obstetric and surgery hospitalisations, psychiatric hospitalizations, home care hospitalizations and post-care and rehabilitation stays with the date and duration, medical procedures and cost coding system. The hospital discharge summary includes the medical unit summaries when the patient is hospitalized successively in several medical units.
  primary diagnosis is the health problem that motivated the admission in the hospital. It is determined at hospital discharge. For patients hospitalized successively in several medical units, the primary diagnosis of the hospitalisation, as well as all medical unit primary diagnoses, are generally taken into account to define the occurrence of an outcome in a pharmacoepidemiology study;
  a linked diagnosis can exist only if the main diagnosis is a care procedure with a code Z of the ICD10 classification (e.g. chemotherapy session) for a chronic or LTD disease. It indicates the pathology at the origin of the care procedure. Linked diagnoses can be used to define chronic diseases but are generally not taken into account to define the occurrence of an outcome in a pharmacoepidemiology study (most being false positives for the studied outcome);
  associated diagnoses are specified if they represent specific healthcare resources. They are mainly underlying chronic diseases. Associated diagnoses can be used to define chronic diseases but are generally not taken into account to define the occurrence of an outcome in a pharmacoepidemiology study (most being false positives for the studied outcome).
  These data are made available in the third quarter following the year considered: i.e. in the third quarter of 2015 for the extraction of 2014 and previous years. The access to the SNIIRAM is regulated and needs approval from the “*Institut national des Données de Santé*” (INDS, National Institute of Healthcare data) and the “*Commission Nationale Informatique et Libertés*” (CNIL, the French data protection commission).
  Database extraction criteria must be fully described in a Data Extraction Plan (DEP) approved prior to initiating extraction. Data are extracted by SNIIRAM data manager and made available on a dedicated portal, or transmitted on encrypted hard drives.

## Analysis variables

### Data extracted from SNDS

To define the study population, the hospital records indicating ACS (primary diagnosis ICD-10 codes I20.0 and I21) were extracted from 1 January 2013 to 31 December 2013;

For the study population, the following data were extracted for the years 2012, 2013 and 2014:

- demographic data: gender, year of birth, month and year of death (for those concerned), CMU-c, residence area;
- LTD status with LTD 30 code, associated ICD-10 code, starting and ending date;
- hospitalisations with admission and discharge dates, duration of stay, ICD-10 codes diagnosis (primary, related and associated), GHM codes, in-hospital procedures (CCAM codes, TNB codes, LPP codes), department of stay, destination after discharge and external consultations;
- treatment reimbursement with date of dispensing, long name, CIP codes, ATC codes, number of packs/units dispensed and dosage, as well as speciality of the prescriber and date of prescription;
- medical visits with date, type, physician specialty, type of activity (private / public); - non-hospital medical procedures with date, number and type of procedure (CCAM code); - lab tests with date, number and type of test (TNB code);
- paramedical consultations and acts (nurse, physiotherapist, orthophonist …) with date, specialty, number of acts;
- medical products and services with date, number and type of products (LPP code); - medical transport with date, type of transport (ambulance, taxi…);
- pension, daily and disability allowances paid by health insurance regimens with date, type, duration;
-other outpatient healthcare with type and date. With this frame, all patients included in 2013 had a one-year history and at least one
  year of follow-up. Bordeaux PharmacoEpi, CIC Bordeaux CIC1401 Confidential 54/799

### Derived variables

For each patient of the study population, the following variables were created:

- **index ACS hospitalisation**. This was defined as the first hospital record indicating ACS (primary diagnosis ICD-10 codes I20.0 or I21) with start and end date between 1 st January 2013 and 31 st December 2013;
- **index date**. This was the index ACS hospitalisation date of discharge;
- **APA treatment exposure**. This was defined by the first dispensing with ATC code of anti-platelet agents excluding heparin (ATC code B01AC) reimbursed between the date of discharge of the index ACS hospitalisation (index date) and the end of follow-up. This indicator was calculated for each APA (ATC codes listed in Appendix E). APA treatments with less than 10 % of exposure was collected in the category “Other APA”;
- **number of days between index date and first dispensing of APA**. This was equal to the difference between index date and date of the first dispensing of APA. This indicator was also categorised into categories according to its distribution;
- **age at index ACS hospitalisation (in years)**. This was equal to the difference between year of birth and year of index date. This indicator was also categorised into categories according to its distribution;
- **coverage by CMU-c at index ACS hospitalisation.** This had the value “yes” if at least one reimbursed healthcare with a CMU-c status had occurred in the six months prior to index date. Otherwise, this had the value “no”;
- **primary diagnosis of the index ACS hospitalisation**. This was defined by the ICD-10 code of the primary diagnosis of the index ACS hospitalisation;
- **at least one stay in an intensive care unit during index ACS hospitalisation**. This was equal to “yes” if at least one stay in the following medical units occurred during the index ACS hospitalisation:
  resuscitation unit (pediatric or not);
  intensive care unit (neurovascular or not). Otherwise, this was equal to “no”;
- **number of days in an intensive care unit during index hospitalisation**. This was equal to the sum by patients of the total number of days of the different stays in an intensive care unit during the index ACS hospitalisation;
- **category of hospital of the index ACS hospitalisation**. This was equal to the following categories:
  teaching hospital - regional hospital; hospital; private hospital.
  Otherwise, patients were excluded;
- **at least one of the following associated diagnosis during index ACS hospitalisation**. This had the value “yes” if at least one associated diagnosis was declared during the index ACS hospitalisation. Otherwise, this had the value “no”. This indicator was created for all and for each associated diagnosis according to the ICD-10 classification;
- **at least one of the following medical procedure performed during index ACS hospitalisation**. This had the value “yes” if at least one medical procedure was performed during the index ACS hospitalisation. Otherwise, this had the value “no”. This indicator was calculated for all and for each associated medical procedure according to the CCAM classification;
- **at least one LTD declared before index ACS hospitalisation**. This had the value “yes” if at least one LTD was declared with a start date before the date of admission of the index ACS hospitalisation. Otherwise, this had the value “no”. This indicator was calculated for all and for each LTD (LTD 1–30, and LTD 99);
- **at least one hospitalisation in the year before index ACS hospitalisation**. This had the value “yes” if at least one hospitalisation occurred in the 365 days before the date of admission of the index ACS hospitalisation. Otherwise, this had the value “no”. This indicator was created for each primary diagnosis according to the ICD-10 code classification;
- **number of hospitalisations per patient in the year before index ACS hospitalisation**. This was equal to the sum by patients of all the hospitalisations occurred in the 365 days before the date of admission of the index ACS hospitalisation. This indicator was categorised according to its distribution;
- **at least one dispensing of treatment in the year before index ACS hospitalisation**. This was equal to “yes” if at least one dispensing of treatment had been reimbursed in the 365 days before the date of admission of the index ACS hospitalisation. Otherwise, this had the value “no”. This indicator was created for each of the following treatments:
  ATC level 1 (first character of ATC code); ATC level 3 (the first four characters of ATC code); ATC level 5 (the seven characters of ATC code) for drug of interest;
- **number of units of a treatment reimbursed in the year before index ACS hospitalisation**. This was equal to the total number of units per box multiplied by the total number of boxes reimbursed during the 365 days before the date of admission of the index ACS hospitalisation for a considered treatment. This indicator was calculated for each APA (ATC codes listed in Appendix E);
- **theoretical coverage period (in days) of a treatment in the year before index ACS hospitalisation**. This was equal to the total number of units reimbursed during the 365 days before the date of admission of the index ACS hospitalisation (under the assumption of one unit covered one day) for a considered treatment. This indicator was calculated for each APA (ATC codes listed in Appendix E);
- **medication possession ratio (MPR) in the year before index ACS hospitalisation**. This was equal to the ratio between the theoretical coverage period (in days) of a treatment during the 365 days before the date of admission of the index ACS hospitalisation and 365 days (duration of the period). This indicator was estimated in percentage and was categorized in seven categories: 0 %,]0-20[%, [20- 40[%, [40-60[%, [60-80[%, [80-100[%, 100 %. This indicator was calculated for each APA;
- **at least one comorbidity in the year before index ACS hospitalisation**. This was to “yes” if at least one history (LTD, primary or related or associated diagnosis in hospital records, *Groupe Homogène de Malade* (GHM), *Groupe Homogène de Séjour* (GHS), medical procedure, specific drug reimbursement) of comorbidity had occurred in the 365 days before the date of admission of the index ACS hospitalisation. Otherwise, this was equal to “no”. This indicator was created for the following cardiac risk factors (relevant ICD10 codes can be provided upon request):
  **acute coronary syndrome (ACS),** defined as a hospital-discharge summary with primary, related or associated diagnosis of ACS;  
  **ischemic or undefined stroke,** defined as an LTD for stroke or a hospital- discharge summary with primary, related or associated diagnosis of stroke;  
  **major bleeding,** defined as a hospital-discharge summary with primary, related or associated diagnosis of bleeding;  
  **coronary artery disease (CAD),** defined as and LTD or a hospital-discharge summary with primary, related or associated diagnosis of CAD;  
  **peripheral arterial disease (PAD),** defined as an LTD for PAD or a hospital- discharge summary with primary, related or associated diagnosis or PAD;  
  **diabetes mellitus**, defined as an LTD for diabetes or a hospital-discharge summary with primary, related or associated diagnosis of diabetes, or ≥ 3 dispensings of anti-diabetic drug excluding benfluorex or ≥ 2 dispensings of anti-diabetic drug with at least one large packaging (box ≥ 80 tablets) during the study period;  
  **hypertension,** defined as an LTD for hypertension or a hospital-discharge summary with primary, related or associated diagnosis of hypertension;  
  **congestive heart failure,** defined as an LTD for heart failure or hospital- discharge summary with primary, related or associated diagnosis of heart failure;  This indicator was also created for the following other comorbidities:  
  **abnormal renal function,** defined as an LTD for renal failure or a hospital- discharge summary with primary, related or associated diagnosis or GHM with abnormal renal function information or dialysis information;
  **abnormal liver function**, defined as an LTD for hepatic disease or a hospital-discharge summary with primary, related or associated diagnosis of hepatic disease;  
  active **cancer**, defined as an LTD for cancer or a hospital-discharge summary with primary, related or associated diagnosis or GHM of cancer, or ≥ 3 dispensings of anti-androgens drug during the study period;  
  **chronic obstructive pulmonary disease (COPD),** defined as defined as an LTD for pulmonary disease or a hospital-discharge summary with primary, related or associated diagnosis of pulmonary disease;  
- **time to follow-up (in days)**. This was equal to the number of days between the date of discharge of the index ACS hospitalisation and the end of follow-up, censored after 365 days. This indicator was also calculated in months and in years;
-**at least one hospitalisation during follow-up period**. This was equal to “yes” if at least one hospitalisation occurred between the date of discharge of the index ACS hospitalisation and the end of follow-up. Otherwise, this was equal to “no”. This indicator was created for:  
  all hospitalisations;
  hospitalisations for ACS (primary diagnosis ICD-10 codes I20.0 and I21);
  hospitalisations for stroke (primary diagnosis ICD-10 codes listed in Appendix B);
  hospitalisations for PCI or CABG (CCAM codes listed in Appendix D);
  hospitalisations for bleeding (primary diagnosis ICD-10 codes listed in Appendix C);
  hospitalisations for ACS, stroke, PCI or CABG;
  hospitalisations for other than ACS, stroke, PCI, CABG or bleeding;
-**number of hospitalisations per patient during follow-up period**.
  This was to the sum by patients of all the hospitalisations that occurred between the date of discharge of the index ACS hospitalisation and the end of follow-up. This indicator was categorised according to its distribution and was created for:  
  all hospitalisations;
  hospitalisations for ACS (primary diagnosis ICD-10 codes I20.0 and I21);
  hospitalisations for stroke (primary diagnosis ICD-10 codes listed in Appendix B);
  hospitalisations for PCI or CABG (CCAM codes listed in Appendix D);
  hospitalisations for bleeding (primary diagnosis ICD-10 codes listed in Appendix C);
  hospitalisations for ACS, stroke, PCI or CABG;
  hospitalisations for other than ACS, stroke, PCI, CABG or bleeding;
-**hospitalisation total duration during follow-up period (in days)**.
  This was equal to the sum by patient of the duration of stay of all the hospitalisations occurred between the date of discharge of the index ACS hospitalisation and the end of follow- up. This indicator was categorized according to its distribution and was created for:  
  all hospitalisations;
  hospitalisations for ACS (primary diagnosis ICD-10 codes I20.0 and I21);
  hospitalisations for stroke (primary diagnosis ICD-10 codes listed in Appendix B);
  hospitalisations for PCI or CABG (CCAM codes listed in Appendix D);
  hospitalisations for bleeding (primary diagnosis ICD-10 codes listed in Appendix C);
  hospitalisations for ACS, stroke, PCI or CABG;
  hospitalisations for other than ACS, stroke, PCI, CABG or bleeding;
-**at least one of the following primary diagnosis during follow-up period**.
  This was equal to “yes” if at least one primary diagnosis had been declared between the date of discharge of the index ACS hospitalisation and the end of follow-up. Otherwise, this was equal to “no”. This indicator was calculated for each primary diagnosis according to the ICD-10 classification;  
- **type of practitioner. For each medical visit,** this was equal to “general practitioner” if its medical specialty was “general medicine” coupled with “practitioner with liberal activities" for the type of activity and “specialist” otherwise;  
- **at least one medical visit during follow-up period**. This was equal to “yes” if at least one medical visit occurred between the date of discharge of the index ACS hospitalisation and the end of follow-up. Otherwise, this was equal to “no”. This indicator was created for all and for each medical speciality of visit practitioner;
- **number of medical visits per patient during follow-up period**. This was equal to the sum by patients of all the medical visits realised between the date of discharge of the index ACS hospitalisation and the end of follow-up. This indicator was categorised according to its distribution and was created for all and for each medical speciality of visit practitioner;
- **at least one lab test during follow-up period**. This was equal to “yes” if at least one lab test had been realised between the date of discharge of the index ACS hospitalisation and the end of follow-up. Otherwise, this was equal to “no”. This indicator was calculated for each lab test according to the *Nomenclature des Actes de Biologie Médicale (*NABM, French medical classification for lab tests) classification;
- **number of lab tests per patient during follow-up period**. This was equal to the sum by patients of all the lab tests realised between the date of discharge of the index ACS hospitalisation and the end of follow-up. This indicator was categorized according to its distribution;
- **at least one dispensing of treatment during follow-up period**. This was equal to “yes” if at least one dispensing of treatment had been reimbursed between the date of discharge of the index ACS hospitalisation and the end of follow-up. Otherwise, this was equal to “no”. This indicator was created for each of the following treatments:
  ATC level 1 (first character of ATC code); ATC level 3 (the first four characters of ATC code); ATC level 5 (the seven characters of ATC code) for drug of interest;
- **number of units of a treatment reimbursed during follow-up period**. This was equal to the total number of units per box multiplied by the total number of boxes reimbursed between the date of discharge of the index ACS hospitalisation and the end of follow-up for a considered treatment. This indicator was calculated for each APA (ATC codes listed in Appendix E);
-**theoretical coverage period (in days) of a treatment during follow-up period**. This was equal to the total number of units reimbursed between the date of discharge
  of the index ACS hospitalisation and the end of follow-up (under the assumption of one unit covered one day) for a considered treatment. This indicator was calculated for each APA (ATC codes listed in appendix);
- **medication possession ratio (MPR) during follow-up period**. This was equal to the ratio between the theoretical coverage period (in days) of a treatment between the date of discharge of the index ACS hospitalisation and the end of follow-up and the duration of the follow-up period. This indicator was estimated in percentage and was categorised in seven categories: 0 %,]0-20[%, [20-40[%, [40-60[%, [60-80[%, [80- 100[%, 100 %. This indicator was calculated for each APA (ATC codes listed in appendix);
- **time to the first dispensing of APA during follow-up period (in days)**. This was equal to the number of days between the date of discharge of the index ACS hospitalisation and the date of the first dispensing of anti-platelet agents excluding heparin (ATC code B01AC) before the end of follow-up. This indicator was also calculated in months and was categorised according to its distribution;
- **at least one co-prescription of other treatment than APA at the first dispensing of APA during follow-up period**. This was equal to “yes” if at least one other treatment than anti-platelet agents excluding heparin (ATC code B01AC) had been prescribed with the same prescription date than the first reimbursement of anti- platelet agents (excluding heparin) after discharge of the index ACS hospitalisation. Otherwise, this was equal to “no”.

This indicator was created for each of the following treatments:

ATC level 1 (first character of ATC code);

ATC level 3 (the first four characters of ATC code);

- **number of dispensings of APA per patient during follow-up period**. This was equal to the sum by patients of all the dispensings of anti-platelet agents excluding heparin (ATC code B01AC) reimbursed between the date of discharge of the index ACS hospitalisation and the end of follow-up. This indicator was categorised according to its distribution;
- **number of different ATC codes of APA per patient during follow-up period**. This was equal to the sum by patients of all the different dispensing with ATC codes of anti-platelet agents excluding heparin (ATC code B01AC) reimbursed between the date of discharge of the index ACS hospitalisation and the end of follow-up. This indicator was categorised according to its distribution;
- **duration of APA treatment dispensing**. This was estimated using the number of packs of APA delivered, the number of units per pack and the defined daily dose (DDD) for the APA;
- **at least one discontinuation of APA treatment during follow-up period**. This was equal to “yes” if at least one gap of 30 days (or more) without dispensing of APA treatment had been observed after the duration (taking into account the supplies of medications of the previous dispensings) of a dispensing of anti-platelet agents excluding heparin (ATC code B01AC) between the dispensing date of the first anti- platelet agents excluding heparin reimbursement and the end of follow-up. Otherwise, this was equal to “no”;
- **date of first discontinuation of APA treatment**. This was equal to the date of the first dispensing of anti-platelet agents excluding heparin (ATC code B01AC) with at least one gap of 30 days (or more) without dispensing of APA after its duration (taking into account the supplies of medications of the previous dispensings) plus its duration;
- **time to the first discontinuation of APA treatment during follow-up period (in days)**. This was equal to the number of days between the date of the first dispensing of anti-platelet agents excluding heparin (ATC code B01AC) and the date of the first discontinuation of APA treatment. This indicator was also calculated in months;
- **at least one discontinuation of initial APA treatment during follow-up period**. This was equal to “yes” if at least one gap of 30 days (or more) without dispensing of initial APA treatment had been observed after the duration (taking into account the supplies of medications of the previous dispensings) of the dispensing of the initial APA between the dispensing date of the initial APA reimbursement and the end of follow-up. Otherwise, this was equal to “no”;
- **date of first discontinuation of initial APA treatment**. This was equal to the date of the first dispensing of initial anti-platelet agents excluding heparin (ATC code B01AC) with at least one gap of 30 days (or more) without dispensing of initial APA after its duration (taking into account the supplies of medications of the previous dispensings) plus its duration;
- **time to the first discontinuation of initial APA treatment during follow-up period (in days)**. This was equal to the number of days between the date of the first dispensing of initial anti-platelet agents excluding heparin (ATC code B01AC) and the date of the first discontinuation of initial APA treatment. This indicator was also calculated in months;
- **at least one switch of initial APA during follow-up period**. This was equal to “yes” if at least one other anti-platelet agents excluding heparin (ATC code B01AC), different from the first APA reimbursed, had been reimbursed between the dispensing date of the first APA and the end of follow-up. Otherwise, this was equal to “no”;
- **time to the first switch of initial APA treatment during follow-up period (in days)**. This was equal to the number of days between the date of the first dispensing of initial anti-platelet agents excluding heparin (ATC code B01AC) and the date of the first dispensing of APA treatment different from the initial APA. This indicator was also calculated in months;
- **at least one retention of initial APA during follow-up period**. This was equal to “yes” if at least one switch or one discontinuation of initial anti-platelet agents excluding heparin (ATC code B01AC) occurred between the dispensing date of the first APA and the end of follow-up. Otherwise, this was equal to “no”;
- **date of retention of initial APA treatment**. This was equal to the date of the first discontinuation of initial anti-platelet agents excluding heparin (ATC code B01AC) or the date of initial APA switch if a switch had occurred before discontinuation;
- **duration of the initial APA exposure (in days)**. This was equal to the number of days between the index date and the date of end of follow-up for patients without initial APA retention, else between the index date and the date of initial APA retention. This indicator was also calculated in months;
- **incident ACS at index ACS hospitalisation**. This was equal to “no” if at least one hospitalisation for ACS (primary diagnosis ICD-10 codes listed in Appendix A) occurred in the 365 days before the date of admission of the index ACS hospitalisation or if at least one LTD for ACS (ICD-10 codes listed in Appendix A) was declared with a start date before the date of admission of the index ACS hospitalisation. Otherwise, this had the value “yes”;
- **naïve of APA treatment at index ACS hospitalisation**. This was equal to “no” if at least one dispensing of APA treatment (excluded ASA) had been reimbursed in the 365 days before the date of admission of the index ACS hospitalisation. Otherwise, this had the value “yes”;
- **incident ACS / naïve of APA treatment at index ACS hospitalisation**. This was equal to “yes” if the indicator “incident ACS at index ACS hospitalisation” was equal to “yes” or/and the indicator “naïve of APA treatment at index ACS hospitalisation” was equal to “yes”. Otherwise, this had the value “no”;
- **Charlson comorbidity index**. This was equal to the sum of the weights of the subjects’ current comorbidities. Comorbid diseases were defined by hospital diagnoses, LTD diagnoses, medical procedures during hospitalisations and drug dispensings during the 365 days prior to index date. The comorbidities and their weights were the following:

With weight = 1: Myocardial infarction; congestive heart failure; peripheral vascular disease; cerebrovascular disease; dementia; chronic pulmonary disease; connective tissue disease; ulcer disease; mild liver disease; diabetes (weight equal to 1);

With weight = 2 or more: diabetes with end-organ damage (weight = 2); moderate or severe renal disease (weight = 2); hemiplegia (weight = 2); any tumour (including leukemia and lymphoma) (weight = 2); moderate or severe liver disease (weight = 3) metastatic solid tumour (weight = 6);

HIV/AIDS (weight equal to 6). Bordeaux PharmacoEpi, CIC Bordeaux CIC1401 Confidential 65/799

To take age into account, each decade after the age of 50 years had a weight of 1. This indicator was categorised in five categories: [0–1], [2–3], [4–5], [6–7], >7;

- **at least one exposure to BASI during initial APA exposure period**. This was equal to “yes” if at least one dispensing of beta-blockers (ATC code C07) or ASA (ATC codes B01AC06 and B01AC30) or statins (ATC codes C10AA, C10BA and C10BX) or ACEI or ARBs (ATC code C09) had been reimbursed between the date of discharge of the index ACS hospitalisation and the end of the initial APA exposure. Otherwise, this was equal to “no”;
- **death during exposure period**. This was equal to “yes” if all-cause death had been declared with a date between the date of discharge of the index ACS hospitalisation and the end of the initial APA exposure. Otherwise, this was equal to “no”;
- **time to death during exposure period (in days)**. This was equal to the number of days between the date of discharge of the index ACS hospitalisation and the date of death. This indicator was also calculated in months;
- **time to hospitalisation during exposure period (in days)**. This was equal to the number of days between the date of discharge of the index ACS hospitalisation and the date of first hospitalisation during the initial APA exposure period. This indicator was calculated in months, categorised according to its distribution and created for:
  hospitalisations for ACS (primary diagnosis ICD-10 codes I20.0 and I21);
  hospitalisations for ACS (primary diagnosis ICD-10 codes I20.0 and I21) in an intensive care unit;
  hospitalisations for stroke (primary diagnosis ICD-10 codes listed in Appendix B);
  hospitalisations for PCI or CABG (CCAM codes listed in Appendix D);
  hospitalisations for bleeding (primary diagnosis ICD-10 codes listed in Appendix C);
- **hospitalisation for ACS or stroke or death during exposure period**. This was equal to “yes” if at least one hospitalisation for ACS or stroke had occurred, or a date of death had been declared between the date of discharge of the index ACS hospitalisation and the end of the initial APA exposure period. Otherwise, this was equal to “no”;
- **time to hospitalisation for ACS or stroke or death during exposure period (in days)**. This was equal to the number of days between the date of discharge of the index ACS hospitalisation and the date of the first following event:
  hospitalisation for ACS; hospitalisation for stroke; death;
  This indicator was also be calculated in months;
- **hospitalisation for ACS, stroke, PCI or CABG or death during follow-up period**. This was equal to “yes” if at least one hospitalisation for ACS, stroke, PCI or CABG had occurred, or a date of death had been declared between the date of discharge of the index ACS hospitalisation and the end of the initial APA exposure period. Otherwise, this was equal to “no”;
- **time to hospitalisation for ACS, stroke, PCI or CABG or death during exposure period (in days)**. This was equal to the number of days between the date of discharge of the index ACS hospitalisation and the date of the first following event:
  hospitalisation for ACS; hospitalisation for stroke; hospitalisation for PCI or CABG; death;
  This indicator was also be calculated in months;
- **hospitalisation for ACS in an intensive care unit or stroke or death during exposure period**. This was equal to “yes” if at least one hospitalisation for ACS in an intensive care unit or one hospitalisation for stroke had occurred, or a date of death had been declared between the date of discharge of the index ACS hospitalisation and the end of the initial APA exposure period. Otherwise, this was equal to “no”;
- **time to hospitalisation for ACS in an intensive care unit or stroke or death during exposure period (in days)**. This was equal to the number of days between the date of discharge of the index ACS hospitalisation and the date of the first following event:
  hospitalisation for ACS in an intensive care unit;
  hospitalisation for stroke;
  death; This indicator was also calculated in months;
- **survival time (in days)**.

For each endpoint:

hospitalisation for ACS or stroke or death; hospitalisation for ACS, stroke, PCI or CABG or death; hospitalisation for ACS in intensive care unit or stroke or death; hospitalisation for bleeding; death; hospitalisation for ACS; hospitalisation for stroke; hospitalisation for PCI or CABG, this was equal to the number of days:

between the date of discharge of the index ACS hospitalisation and considered endpoint date for the patients with an endpoint;

From the date of discharge of the index ACS hospitalisation to the end of the initial APA exposure, loss to follow-up or censoring for patients without endpoints;

### Data handling

The following decision-making rules will be applied:

- for dates, if the year and month was provided but not the day, then the day was set at 15th of the month. Specifically concerning the date of death (which has no day provided), if an event or hospitalisation occurred the same month after the 15th, the date of event/end of hospitalisation was chosen;
- for the estimation of the MPR, values > 100 % was set to 100 %;
- for the number of units reimbursed in the 365 days before the date of admission of the index ACS hospitalisation, in the case of dispensings before index date which could cover some days after index date, only days before index date was considered;
- for the number of units reimbursed during the follow-up period, in the case of dispensings before the end of follow-up which could cover some days after the end of follow-up, only days before the end of follow-up was considered;
- for the number of lab tests occurred during follow-up period, all the tests made the same day was considered like one lab test;

## Statistical analysis

### Populations and variables analysed

A flow chart depicting the total number of patients in the database satisfying the extraction criteria and the number of patients for the study population will be prepared.

The following analyses were performed for the whole population and according to the different APA prescribed after discharge (including no APA treatment):

- description of the characteristics of patients hospitalised for ACS:
  initial APA treatment;
  gender, age at inclusion, CMU-c;
  index ACS hospitalisation using main diagnosis, procedure performed during hospitalisation, hospitalisation duration;
- description of the one-year period prior to index date:
  history of cardiac diseases using LTD, diagnosis of previous hospitalisations, related and associated diagnoses to index ACS hospitalisation;
  history of dispensings of drugs;
  cardiac risk factors using LTD, diagnosis of previous hospitalisations, related and associated diagnoses to index ACS hospitalisation and one-year specific drug reimbursement history;
  other comorbidities using LTD, related and associated diagnoses to index ACS hospitalisation, one-year history of hospitalisations and one-year other drug reimbursement history;
- description of the one-year period after index date:
  hospitalisations with details of diagnoses;
  medical visits, with details of physician speciality;
  lab tests, with details of type of test;
  dispensings of all drugs;
  usage patterns of each APA: frequency, quantities, MPR, co-prescription, duration and persistence (discontinuation, switches, retention rates).

The following analyses were performed for the whole population and for the matched population according to the initial APA treatment (Clopidogrel, Prasugrel and Ticagrelor) and according to the diagnosis of the index hospitalisation:

- description of the usage patterns of APA (discontinuation, switches, retention rates);  
- estimation of the probability of receiving initial APA treatment (Ticagrelor vs. Clopidogrel and Ticagrelor vs. Prasugrel) using a hdPS;
- distribution of hdPS according to comparison groups; 
- comparison of the patients’ characteristics between initial APA treatment groups using standardised differences; 
- estimation of the one-year incidence of endpoints during initial APA exposure period:
  primary effectiveness endpoint: a composite of hospitalisation for ACS (primary diagnosis ICD-10 codes I20. and I21), hospitalisation for an ischemic or undefined stroke (primary diagnosis ICD-10 codes listed in Appendix B) and all-cause death;
  secondary effectiveness endpoint: a composite of hospitalisation for ACS (primary diagnosis ICD-10 codes I20. and I21), hospitalisation for an ischemic or undefined stroke (primary diagnosis ICD-10 codes listed in Appendix B), hospitalisation for PCI or CABG (CCAM codes listed in Appendix D) and all- cause death;
  secondary effectiveness endpoint: a composite of hospitalisation for ACS (primary diagnosis ICD-10 codes I20. and I21) in an intensive care unit, hospitalisation for an ischemic or undefined stroke (primary diagnosis ICD-10 codes listed in Appendix B) and all-cause death;
  safety endpoint: hospitalisation for a major bleeding, including hemorrhagic stroke (primary diagnosis ICD-10 codes listed in Appendix C);
  all-cause death;
  hospitalisation for ACS;
  hospitalisation for an ischemic or undefined stroke;
  hospitalisation for PCI or CABG;
  hospitalisation for ACS in an intensive care unit;
- estimation of the one-year cumulative incidence of all the endpoints during initial APA exposure period;
- comparison of the one-year incidence of all the endpoints between initial APA treatment groups:
  crude analysis;
  analysis adjusted on APA hdPS, gender, age, ASA associated to APA at dispensing index date, incident ACS/naïve APA and exposure to BASI during initial APA exposure period;
  matched analysis adjusted on ASA associated to APA at dispensing index date, incident ACS/naïve APA and exposure to BASI during initial APA exposure period.

### Analytical method

Statistical analysis was conducted by the Department of Pharmacology of Bordeaux, using SAS® software (SAS Institute, Version 9.4, North Carolina, USA).

#### Descriptive analysis

The descriptive analysis of qualitative and ordinal variables were presented using the frequency and the proportion of each modality, including missing data as modality. In stratified analyses, the percentages and distributions were estimated in columns in each stratum.

Quantitative variables were presented using the size, number of patients with missing data, the arithmetic mean, standard deviation, the first quartile, median, third quartile, and extreme values. Graph (flowchart, histogram) were used to present the selection of patients.

The SNIIRAM database covers actually more than 95 % of the French population. As the study population is not a sample, two-sided tests and 95 % confidence intervals (CIs) was not performed. Standardized differences were presented to assess size effects between treatment groups in crude, adjusted and matched analysis.

#### Comparisons

The comparisons between the treatment groups (according to index diagnosis) for all and matched patients were performed using crude (unadjusted), stratified and weighted (adjusted) standardised differences to assess size effects between groups. Unadjusted standardised differences (Austin and Hamdani, 2006) were evaluated from the formulas:

- for continuous variables:

where X1 and X2 denote the sample mean of a variable in each group and s12 and s22 denote the sample variances, respectively;

- for binary categorical variables:

where p1 and p2 denote the proportion of a binary variable in each group, respectively.

For stratified analysis, standardised differences were evaluated into five approximately equal-size groups using the quintiles of the hdPS.

For weighted analysis, standardised differences were evaluated from predicted values of a regression model (linear model regression for continuous variables and logistic regression model for dichotomous variables). In the regression model, the variable to be compared was regressed with hdPS, treatment group and interaction between hdPS and treatment group.

#### High dimensional propensity score

The probability to be treated by individual APA (Ticagrelor vs. Clopidogrel and Ticagrelor vs. Prasugrel) after the index ACS hospitalisation (according to index diagnosis) was estimated using a hdPS. The hdPS algorithm (Rassen et al., 2011) grounds on automated technique (available in a SAS® macro) that examines thousands of covariates among different claims data dimensions in the study population.

$$\begin{array}{cc} \text{d}=\frac{\left(\hat{P_{1}}-\hat{P_{2}}\right)}{\sqrt{\frac{\left[\hat{P_{1}}\left(1-\hat{P_{1}}\right)+\hat{P_{2}}\left(1-\hat{P_{2}}\right)\right]}{2}}} & \text{d}=\frac{\left(\bar{X_{1}}-\bar{X_{2}}\right)}{\sqrt{\frac{S_{1}^{2}+S_{2}^{2}}{2}}} \end{array}$$

Each dimension describes an aspect of care and common data dimensions include patient characteristics (age, gender, CMU-c, index PCI, index CABG, duration of the index hospitalisation, at least one dispensing of ASA before index date, category of hospital of the index hospitalisation, intensive care during index hospitalisation, incident ACS/naïve APA, Charlson comorbidity index), history of disease (LTD), hospitalisation (diagnoses), healthcare reimbursement (medical or paramedical visits and lab tests) and drug dispensing (See Appendix F).

From each dimension, the top *n* most prevalent codes are transformed into binary covariates according to three levels of within-patient frequency of occurrence:

- once (occurred greater than or equal to one time); 
- sporadic (occurred greater than or equal to the median number of times the code is observed);
- frequent (occurred greater than or equal to the 75th percentile number of times the code is observed).

These binary covariates are then individually assessed for selection into a propensity score using a logistic regression model. The algorithm prioritizes each of these variables by its potential to bias the exposure-outcome relationship (Bross’s bias formula, 1966) and includes by default the top 500 of these covariates in the propensity score.

The APA hdPS distributions were examined to check that the overlap between APA is correct to perform matched analysis (1:1 nearest neighbour matching was used), as well as to exclude outliers (extreme values of hdPS) in adjusted analysis.

#### Matching

For each comparison (Ticagrelor vs. Clopidogrel and Ticagrelor vs. Prasugrel), a subsample was selected within each treatment group using a 1:1 matching algorithm to match couples of treatment groups on:

- gender;
- age at index date +/-1 year;
- hdPS +/-0.05;
- index diagnosis (UA, STEMI, NSTEMI).

The nearest neighbor, optimal and greedy matching algorithms were used to match patients in either study group, until none of the remaining patients could be matched.

#### Incidence estimations

Primary and secondary endpoints were analysed using survival methods:

- Incidence in person-years (PY) was estimated using total number of person-years that patients at risk contributed during initial APA exposure period as a denominator, and the number of events occurring during initial APA exposure period as a numerator;
- Kaplan-Meier estimate for cumulative incidence of endpoints. For each endpoint, the first occurrence was considered during the initial APA exposure period;
- Cox proportional hazard risk model to compare incidence between APA; - Poisson or quasi-Poisson regression model to compare incidence between APA when number of events were lower or equal to 100.

For the Cox models, time proportional hazard assumption were checked for each covariate tested in model. Results were expressed as hazard ratios (HR) and 95 % CI and Wald test p-value. The confounding between variables were investigated.

## Validation of method; quality of matching

**Fig. 1:**
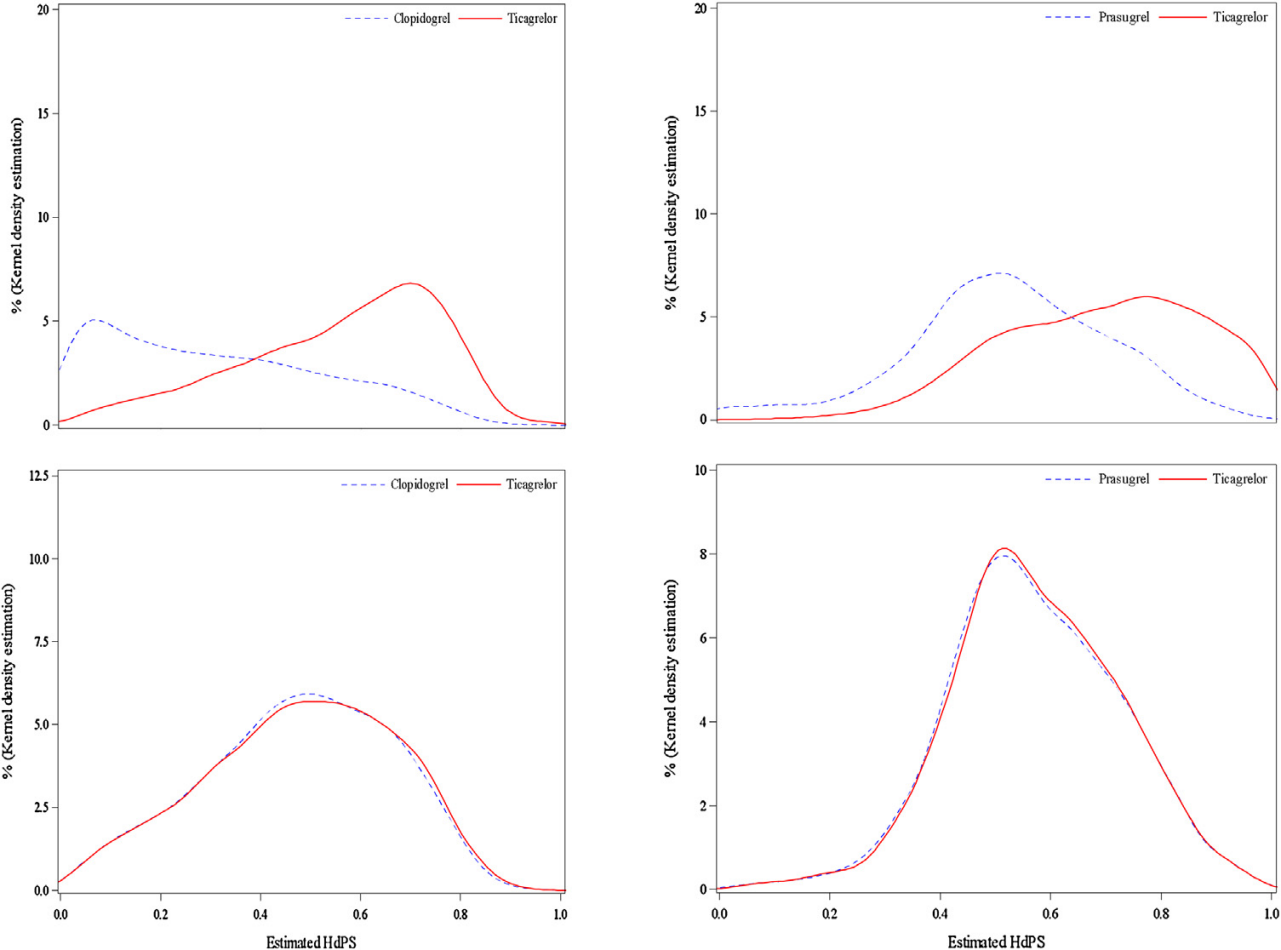
distribution of unmatched (upper panels) and matched (lower panels) high-dimensional propensity scores for ticagrelor vs. clopidogrel (left panels) and ticagrelor vs. prasugrel (right panels).

The distributions of the high-dimensional propensity scores in the crude population and in the cohorts obtained after matching are shown in Fig. 1 for ticagrelor vs. clopidogrel and for ticagrelor vs. prasugrel.

**Fig. 2:**
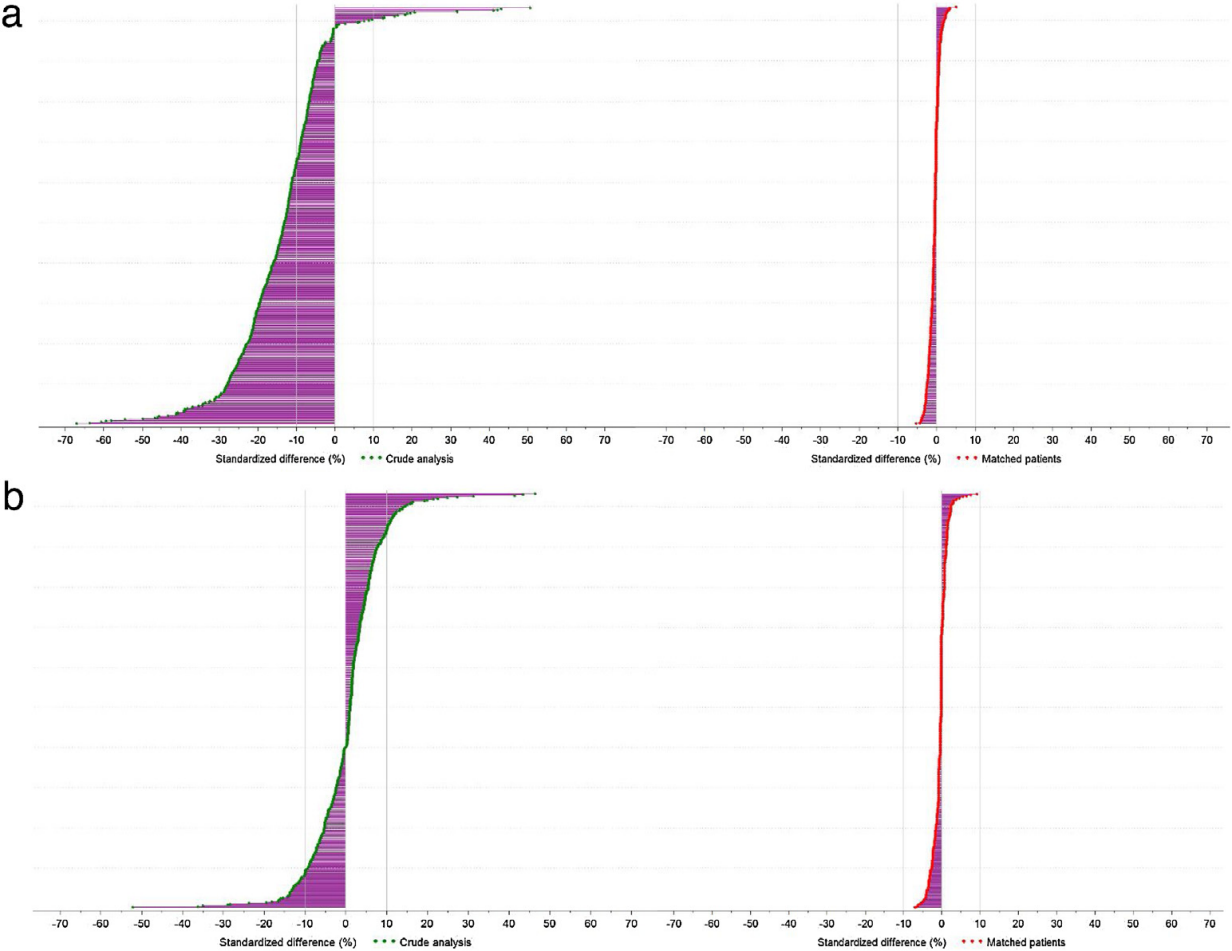
(a) Standardized differences in characteristics (n = 516) included in the hdPS (before and after matching): Ticagrelor vs. Clopidogrel. (b) Standardized differences in characteristics (n = 516) included in the hdPS (before and after matching): Ticagrelor vs. Prasugrel.

Fig. 2 shows the distribution of standardized mean differences between the unmatched and matched populations, for ticagrelor vs. clopidogrel (2a) and for ticagrelor vs. prasugrel (2b) for over 500 population variables.

### In conclusion

In this large population database that is newly accessible, the use of high-dimensional propensity score matching on large numbers of routinely collected variables results in essentially identical populations on over 500 parameters, including direct or indirect potential confounders.

## Funding and declaration of competing interest

This paper describes the methods used in a study of post-myocardial infarction use of antiplatelet agents. This study was requested by the French National Authorities and funded by Astra-Zeneca. Among the authors of the present methods paper, Nicholas Moore is a tenured employee of University of Bordeaux, state funded. The other authors are employees of Bordeaux PharmacoEpi, a research platform of University of Bordeaux. Their salaries are derived from the payments made by the sponsors of this study and all the other studies done by the platform, with public or private funding. In the present paper, there is no mention of the effects of any medicinal product, and no conflict of interest.

## Figures and Tables

**Table:** 

**ICD-10 code**	**ICD-10 label**
I20.0	Unstable angina

**I21**	**Acute myocardial infarction**
I21.0	Acute transmural myocardial infarction of anterior wall
I21.1	Acute transmural myocardial infarction of inferior wall
I21.2	Acute transmural myocardial infarction of other sites
I21.3	Acute transmural myocardial infarction of unspecified site
I21.4	Acute subendocardial myocardial infarction
I21.9	Acute myocardial infarction, unspecified

**Table:** 

**ICD-10 code**	**ICD-10 label**
**I63**	**Cerebral infarction**
I63.0	Cerebral infarction due to thrombosis of precerebral arteries
I63.1	Cerebral infarction due to embolism of precerebral arteries
I63.2	Cerebral infarction due to unspecified occlusion or stenosis of precerebral arteries
I63.3	Cerebral infarction due to thrombosis of cerebral arteries
I63.4	Cerebral infarction due to embolism of cerebral arteries
I63.5	Cerebral infarction due to unspecified occlusion or stenosis of cerebral arteries
I63.6	Cerebral infarction due to cerebral venous thrombosis, nonpyogenic
I63.8	Other cerebral infarction
I63.9	Cerebral infarction, unspecified

**I64**	**Stroke, not specified as haemorrhage or infarction**

**Table:** 

**ICD-10 code**	**ICD-10 label**
**D62**	Acute posthaemorrhagic anemia
**D68.3**	Haemorrhagic disorder due to circulating anticoagulants
**D69.8**	Other specified haemorrhagic conditions
**D69.9**	Haemorrhagic condition, unspecified
**H11.3**	Conjunctival haemorrhage
**H31.3**	Choroidal haemorrhage and rupture
**H35.6**	Retinal haemorrhage
**H43.1**	Vitreous haemorrhage
**H45.0**	Vitreous haemorrhage in diseases classified elsewhere
**H92.2**	Otorrhagia
**I23.0**	Haemopericardium as current complication following acute myocardial infarction
**I31.2**	Haemopericardium, not elsewhere classified
**I60.0**	Subarachnoid haemorrhage from carotid siphon and bifurcation
**I60.1**	Subarachnoid haemorrhage from middle cerebral artery
**I60.2**	Subarachnoid haemorrhage from anterior communicating artery
**I60.3**	Subarachnoid haemorrhage from posterior communicating artery
**I60.4**	Subarachnoid haemorrhage from basilar artery
**I60.5**	Subarachnoid haemorrhage from vertebral artery
**I60.6**	Subarachnoid haemorrhage from other intracranial arteries
**I60.7**	Subarachnoid haemorrhage from intracranial artery, unspecified
**I60.8**	Other subarachnoid haemorrhage
**I60.9**	Subarachnoid haemorrhage, unspecified
**I61.0**	Intracerebral haemorrhage in hemisphere, subcortical
**I61.1**	Intracerebral haemorrhage in hemisphere, cortical
**I61.2**	Intracerebral haemorrhage in hemisphere, unspecified
**I61.3**	Intracerebral haemorrhage in brain stem
**I61.4**	Intracerebral haemorrhage in cerebellum
**I61.5**	Intracerebral haemorrhage, intraventricular
**I61.6**	Intracerebral haemorrhage, multiple localized
**I61.8**	Other intracerebral haemorrhage
**I61.9**	Intracerebral haemorrhage, unspecified
**I62.0**	Subdural haemorrhage (acute)(nontraumatic)
**I62.1**	Nontraumatic extradural haemorrhage
**I62.9**	Intracranial haemorrhage (nontraumatic), unspecified
**I85.0**	Oesophageal varices with bleeding
**I98.3**	Oesophageal varices with bleeding in diseases classified elsewhere
**J94.2**	Haemothorax
**K22.6**	Gastro-oesophageal laceration-haemorrhage syndrome
**K25.0**	Gastric ulcer: Acute with haemorrhage
**K25.2**	Gastric ulcer: Acute with both haemorrhage and perforation
**K25.4**	Gastric ulcer: Chronic or unspecified with haemorrhage
**K25.6**	Gastric ulcer: Chronic or unspecified with both haemorrhage and perforation
**K26.0**	Duodenal ulcer: Acute with haemorrhage
**K26.2**	Duodenal ulcer: Acute with both haemorrhage and perforation
**K26.4**	Duodenal ulcer: Chronic or unspecified with haemorrhage
**K26.6**	Duodenal ulcer: Chronic or unspecified with both haemorrhage and perforation
**K27.0**	Peptic ulcer, site unspecified: Acute with haemorrhage
**K27.2**	Peptic ulcer, site unspecified: Acute with both haemorrhage and perforation
**K27.4**	Peptic ulcer, site unspecified: Chronic or unspecified with haemorrhage
**K27.6**	Peptic ulcer, site unspecified: Chronic or unspecified with both haemorrhage and perforation
**K28.0**	Gastrojejunal ulcer: Acute with haemorrhage
**K28.2**	Gastrojejunal ulcer: Acute with both haemorrhage and perforation
**K28.4**	Gastrojejunal ulcer: Chronic or unspecified with haemorrhage
**K28.6**	Gastrojejunal ulcer: Chronic or unspecified with both haemorrhage and perforation
**K29.0**	Acute haemorrhagic gastritis
**K62.5**	Haemorrhage of anus and rectum
**K66.1**	Haemoperitoneum
**K76.2**	Central haemorrhagic necrosis of liver
**K92.0**	Haematemesis
**K92.1**	Melaena
**K92.2**	Gastrointestinal haemorrhage, unspecified
**M25.0**	Haemarthrosis
**N02.0**	Recurrent and persistent haematuria: Minor glomerular abnormality
**N02.1**	Recurrent and persistent haematuria: Focal and segmental glomerular lesions
**N02.2**	Recurrent and persistent haematuria: Diffuse membranous glomerulonephritis
**N02.3**	Recurrent and persistent haematuria: Diffuse mesangial proliferative glomerulonephritis
**N02.4**	Recurrent and persistent haematuria: Diffuse endocapillary proliferative glomerulonephritis
**N02.5**	Recurrent and persistent haematuria: Diffuse mesangiocapillary glomerulonephritis
**N02.6**	Recurrent and persistent haematuria: Dense deposit disease
**N02.7**	Recurrent and persistent haematuria: Diffuse crescentic glomerulonephritis
**N02.8**	Recurrent and persistent haematuria: Other
**N02.9**	Recurrent and persistent haematuria: Unspecified
**N42.1**	Congestion and haemorrhage of prostate
**N92.0**	Excessive and frequent menstruation with regular cycle
**N92.1**	Excessive and frequent menstruation with irregular cycle
**N92.3**	Ovulation bleeding
**N92.4**	Excessive bleeding in the premenopausal period
**N93.0**	Postcoital and contact bleeding
**N93.8**	Other specified abnormal uterine and vaginal bleeding
**N93.9**	Abnormal uterine and vaginal bleeding, unspecified
**N95.0**	Postmenopausal bleeding
**R04.0**	Epistaxis
**R04.1**	Haemorrhage from throat
**R04.2**	Haemoptysis
**R04.8**	Haemorrhage from other sites in respiratory passages
**R04.9**	Haemorrhage from respiratory passages, unspecified
**R31**	Unspecified haematuria
**R58**	Haemorrhage, not elsewhere classified
**S06.4**	Epidural haemorrhage
**S06.5**	Traumatic subdural haemorrhage
**S06.6**	Traumatic subarachnoid haemorrhage
**S26.0**	Injury of heart with haemopericardium
**S27.1**	Traumatic haemothorax
**T79.2**	Traumatic secondary and recurrent haemorrhage

**Table:** 

	CCAM code	CCAM label
**Percutaneous coronary intervention (PCI)**	**DDAA002**	Angioplastie d’élargissement du tronc commun de l’artère coronaire gauche par thoracotomie avec CEC
	**DDAF001**	Dilatation intraluminale d’un vaisseau coronaire sans pose d’endoprothèse par voie artérielle transcutanée
	**DDAF003**	Dilatation intraluminale de 3 vaisseaux coronaires ou plus avec pose d’endoprothèse par voie artérielle transcutanée
	**DDAF004**	Dilatation intraluminale de 2 vaisseaux coronaires avec pose d’endoprothèse par voie artérielle transcutanée
	**DDAF006**	Dilatation intraluminale d’un vaisseau coronaire avec pose d’endoprothèse par voie artérielle transcutanée
	**DDAF007**	Dilatation intraluminale de 2 vaisseaux coronaires avec artériographie coronaire avec pose d’endoprothèse par voie artérielle transcutanée
	**DDAF008**	Dilatation intraluminale d’un vaisseau coronaire avec artériographie coronaire avec pose d’endoprothèse par voie artérielle transcutanée
	**DDAF009**	Dilatation intraluminale de 3 vaisseaux coronaires ou plus avec artériographie coronaire avec pose d’endoprothèse par voie artérielle transcutanée
	**DDAF010**	Dilatation intraluminale d’un vaisseau coronaire avec artériographie coronaire sans pose d’endoprothèse par voie artérielle transcutanée
	**DDFF001**	Athérectomie intraluminale d’artère coronaire par méthode rotatoire [rotationnelle] par voie artérielle transcutanée
	**DDFF002**	Athérectomie intraluminale d’artère coronaire par voie artérielle transcutanée
	**DDLF001**	Injection in situ d’agent pharmacologique dans une branche d’artère coronaire pour réduction de l’épaisseur du septum interventriculaire par voie artérielle transcutanée
	**DDPF002**	Recanalisation d’artère coronaire avec pose d’endoprothèse par voie artérielle transcutanée
	**DDSF001**	Embolisation ou fermeture d’une fistule ou d’un anévrisme coronaire par voie vasculaire transcutanée
	**YYYY082**	Tentative d’angioplastie d’un vaisseau coronaire d’une artère subclavière viscérale ou périphérique en cas de non franchissement de la sténose

**Coronary artery bypass graft (CABG)**	**DDMA003**	Revascularisation coronaire par 3 greffons artériels avec 3 anastomoses distales par thoracotomie avec CEC
	**DDMA004**	Revascularisation coronaire par greffon veineux avec 4 anastomoses distales ou plus par thoracotomie avec CEC
	**DDMA005**	Revascularisation coronaire par 2 greffons artériels et par greffon veineux avec 3 anastomoses distales par thoracotomie avec CEC
	**DDMA006**	Revascularisation coronaire par 2 greffons artériels avec 3 anastomoses distales par thoracotomie avec CEC
	**DDMA007**	Revascularisation coronaire par greffon veineux avec une anastomose distale par thoracotomie avec CEC
	**DDMA008**	Revascularisation coronaire par 2 greffons artériels avec 4 anastomoses distales ou plus par thoracotomie avec CEC
	**DDMA009**	Revascularisation coronaire par 2 greffons artériels et par greffon veineux avec 4 anastomoses distales ou plus par thoracotomie avec CEC
	**DDMA011**	Revascularisation coronaire par un greffon artériel et par greffon veineux avec 2 anastomoses distales par thoracotomie avec CEC
	**DDMA012**	Revascularisation coronaire par 3 greffons artériels et par greffon veineux avec 4 anastomoses distales ou plus par thoracotomie avec CEC
	**DDMA013**	Revascularisation coronaire par 3 greffons artériels avec 4 anastomoses distales ou plus par thoracotomie avec CEC
	**DDMA015**	Revascularisation coronaire par un greffon artériel avec une anastomose distale par thoracotomie avec CEC
	**DDMA016**	Revascularisation coronaire par greffon veineux avec 3 anastomoses distales par thoracotomie avec CEC
	**DDMA017**	Revascularisation coronaire par un greffon artériel avec 2 anastomoses distales par thoracotomie avec CEC
	**DDMA018**	Revascularisation coronaire par un greffon artériel et par greffon veineux avec 3 anastomoses distales par thoracotomie avec CEC
	**DDMA019**	Revascularisation coronaire par greffon veineux avec 2 anastomoses distales par thoracotomie avec CEC
	**DDMA020**	Revascularisation coronaire par 2 greffons artériels avec 2 anastomoses distales par thoracotomie avec CEC
	**DDMA021**	Revascularisation coronaire par un greffon artériel et par greffon veineux avec 4 anastomoses distales ou plus par thoracotomie avec CEC
	**DDMA022**	Revascularisation coronaire par 2 greffons artériels et par greffon veineux avec 3 anastomoses distales par thoracotomie sans CEC
	**DDMA024**	Revascularisation coronaire par greffon veineux avec 2 anastomoses distales par thoracotomie sans CEC
	**DDMA025**	Revascularisation coronaire par un greffon artériel avec une anastomose distale par thoracotomie sans CEC
	**DDMA026**	Revascularisation coronaire par 2 greffons artériels avec 2 anastomoses distales par thoracotomie sans CEC
	**DDMA027**	Revascularisation coronaire par greffon veineux avec 3 anastomoses distales par thoracotomie sans CEC
	**DDMA028**	Revascularisation coronaire par greffon veineux avec une anastomose distale par thoracotomie sans CEC
	**DDMA029**	Revascularisation coronaire par un greffon artériel et par greffon veineux avec 3 anastomoses distales par thoracotomie sans CEC
	**DDMA030**	Revascularisation coronaire par 3 greffons artériels avec 3 anastomoses distales par thoracotomie sans CEC
	**DDMA031**	Revascularisation coronaire par 2 greffons artériels avec 3 anastomoses distales par thoracotomie sans CEC
	**DDMA032**	Revascularisation coronaire par un greffon artériel et par greffon veineux avec 2 anastomoses distales par thoracotomie sans CEC
	**DDMA033**	Revascularisation coronaire par 2 greffons artériels avec 4 anastomoses distales ou plus par thoracotomie sans CEC
	**DDMA034**	Revascularisation coronaire par 2 greffons artériels et par greffon veineux avec 4 anastomoses distales ou plus par thoracotomie sans CEC
	**DDMA035**	Revascularisation coronaire par 3 greffons artériels avec 4 anastomoses distales ou plus par thoracotomie sans CEC
	**DDMA036**	Revascularisation coronaire par 3 greffons artériels et par greffon veineux avec 4 anastomoses distales ou plus par thoracotomie sans CEC
	**DDMA037**	Revascularisation coronaire par greffon veineux avec 4 anastomoses distales ou plus par thoracotomie sans CEC
	**DDMA038**	Revascularisation coronaire par un greffon artériel et par greffon veineux avec 4 anastomoses distales ou plus par thoracotomie sans CEC
	**ENFA003**	Prélèvement peropératoire d’autogreffe artérielle pour pontage vasculaire
	**EPFA006**	Prélèvement peropératoire d’autogreffe veineuse pour pontage vasculaire

**Table:** 

ATC Codes	ATC labels
B01AC01	DITAZOLE
B01AC02	CLORICROMEN
B01AC03	PICOTAMIDE
B01AC04	CLOPIDOGREL
B01AC05	TICLOPIDINE
B01AC06	ACETYLSALICYLIC ACID
B01AC07	DIPYRIDAMOLE
B01AC08	CARBASALATE CALCIUM
B01AC09	EPOPROSTENOL
B01AC10	INDOBUFEN
B01AC11	ILOPROST
B01AC13	ABCIXIMAB
B01AC15	ALOXIPRINE
B01AC16	EPTIFIBATIDE
B01AC17	TIROFIBAN
B01AC18	TRIFLUNISAL
B01AC19	BERAPROST
B01AC21	TREPROSTINIL
B01AC22	PRASUGREL
B01AC23	CILOSTAZOL
B01AC24	TICAGRELOR
B01AC25	CANGRELOR
B01AC26	VORAPAXAR
B01AC30	COMBINATIONS
B01AC56	ACETYLSALICYLIC ACID, COMBINATIONS WITH PROTON PUMP INHIBITORS

## Appendix F Variables used for the high-dimensional propensity score matching

Covariates present at index date:

age, gender, CMU-c (social deprivation indicator)

Index PCI, Index CABG, duration (in days) of the index hospitalization,

Number of days in an intensive care unit during index hospitalization,

Category of hospital of the index hospitalization,

At least one dispensing of ASA before index date,

Incident ACS/naïve APA,

Charlson comorbidity index (in categories)

Empirical variables created from 5 dimensions in previous history (see statistical methods):

1) Chronic diseases (ICD-10 code 3 digits) present before index date,
2) Hospitalization diagnoses (ICD-10 code 3 digits) in the year before index date,
3) Cardiovascular drugs dispensings (ATC code 7 digits) in the year before index date,
4) non-cardiovascular drugs dispensings (ATC code 7 digits) in the year before index date
5) medical or paramedical visits or lab tests (NABM code) in the year before index date

These binary covariates are then individually assessed for selection into a propensity score using a logistic regression model. The algorithm prioritizes each of these variables by its potential to bias the exposure-outcome relationship and includes by default the top 500 of these covariates in the propensity score.
